# A Successful Obstetric Outcome in a Woman With Familial Hypokalemic Periodic Paralysis: Challenges in Management

**DOI:** 10.7759/cureus.30424

**Published:** 2022-10-18

**Authors:** Vignesh Durai, Bhabani Pegu, Murali Subbaiah

**Affiliations:** 1 Obstetrics and Gynaecology, Jawaharlal Institute of Postgraduate Medical Education and Research, Puducherry, IND

**Keywords:** pregnancy outcome, familial hypokalaemia periodic paralysis, periodic paralysis, hypokalaemia, pregnancy

## Abstract

Hypokalemic periodic paralysis during pregnancy is a rare disease condition that presents during pregnancy. It manifests with acute muscular weakness associated with low potassium levels. We report a case of an antenatal woman presenting with leaking per vagina and weakness of four limbs at 33 weeks of gestation. She had similar episodes in the past but defaulted on taking oral potassium. On physical examination, she had hyporeflexia and flaccid paralysis of all limbs without sensory involvement. A neurology consultation was sought and diagnosed to have flaccid quadriparesis. Her investigations showed low serum potassium along with electrocardiography (ECG) changes. With potassium correction, her weakness improved within four days of initiating treatment. A week later, she had a spontaneous labour onset and delivered a healthy male baby. The peripartum period was uneventful. A timely diagnosis and management, avoiding precipitating factors, and preventing future attacks should be the primary goal of management.

## Introduction

Hypokalemic periodic paralysis is an acute reversible condition characterized by episodic weakness or paralysis of proximal muscles. It occurs due to a decrease in the serum potassium level, which could be a primary disorder inherited as autosomal dominant channelopathy of voltage-gated calcium/sodium channels of muscles. The well-known complications of hypokalaemia are life-threatening, such as cardiac arrhythmias and respiratory muscle paralysis, and in severe cases, rhabdomyolysis has also been reported [1]. Pregnancy is known to cause worsening of familial hypokalemia periodic paralysis, but there are no guidelines or protocols for managing a pregnant woman with this condition [2]. Although most cases are genetically inherited, women are rarely affected due to the incomplete penetrance of the genes [3].

## Case presentation

A 26-year-old primigravida was referred with weakness of extremities for two days at 33 weeks of gestation. She also complained of leaking per vagina for one day. She had two similar episodes of limb weakness during her childhood and was diagnosed with a case of hypokalemia paralysis. She was non-compliant with medications and was on intermittent follow-up. She was later found to have familial hypokalemia periodic paralysis as her cousin’s sister had similar episodes and was on potassium supplementation.

On examination, her vitals were within normal range. Neurological examination revealed normal cortical functions with no sensory or cranial nerve involvement. The bulk of the muscles was average in both upper and lower limbs. But there was a proportional decrease in power (Medical Research Council grade 3/5) and hyporeflexia in all extremities. The uterus was 32 weeks, relaxed, and nontender, with a reasonable fetal heart rate. On speculum examination, frank, non-foul-smelling liquor drained. Routine investigations, high vaginal swabs, and urine samples for culture were sent. The non-stress test was reactive, and the obstetric ultrasound revealed a single live fetus of 2.2kg with good liquor. The patient was admitted to the intensive care unit (ICU) and empirically started on antibiotics. A course of dexamethasone was given for fetal lung maturity. A neurological opinion was obtained, and she was found to have flaccid quadriparesis.

Apart from low serum potassium (2.5mEq/L), all electrolytes were normal. Her investigations are summarised in Table 1.

**Table 1:** Summary of Investigations

Laboratory tests	Patient values					Reference range
Haemoglobin (g/dL)	10.2					12.5 – 15.5
Platelet (per cu. mm)	2.2 lacs					1.5 – 4.5 lacs
Creatinine (mg/dL)	0.9					0.5 – 0.9
Sodium (mEq/L)	137					136 - 146
Potassium (mg/dL)	Day 1	Day 2	Day 3	Day 4	Day 5	3.5 – 5.5
	2.5	2.9	3.3	4	4.8	
Calcium (mg/dL)	9.8					9 – 11
Magnesium (mg/dL)	2.6					1.8 – 3
Phosphate (mg/dL)	3.0					2.5 – 4.5
Lactate dehydrogenase (LDH) (IU/L)	184					60 – 200
Creatinine kinase (CK) (IU/L)	55					20 - 170
Thyroid-stimulating hormone (μIU/ml)	1.9					0.35 – 5.5
Urine routine	Normal					
Urine culture	Sterile					
High vaginal swab culture	Sterile					

On electrocardiography (ECG), there was a characteristic flattening of the T wave with a prominent U wave. Therefore, intravenous (IV) potassium correction was started at the dose of 20 mEq of potassium chloride (KCl) in 500ml normal saline over four hours every eight hours for 48 hours. Oral KCl was started (15ml=20 mEq, thrice daily) after 48 hours of intravenous therapy. Within four days of potassium supplementation, her weakness improved. She spontaneously went into labour a week later and had an uneventful intrapartum period. IV opioid was given intrapartum as labour analgesia, and she delivered a 2.2kg healthy male baby by low forceps application due to fetal distress. She was discharged on the fifth postnatal day with oral potassium supplements and biweekly potassium monitoring. The neonate was not evaluated as it usually manifests in late childhood or adolescence.

## Discussion

The most common type of hypokalemia periodic paralysis (hypo-PP) is familial hypokalemia periodic paralysis (f-hypo-PP). Hence, a diagnosis can be established when there is a triad of episodic paralysis, hypokalemia during an attack, and strong family history as it is inherited in an autosomal dominant fashion. Our patient fulfilled all three criteria and hence was diagnosed with f-hypo-PP. The mutation involves calcium channel, voltage-dependent, L type, alpha 1S (CACNA1S) subunit and sodium channel, voltage-gated, type-IV, alpha subunit (SCN4A) genes which encode for calcium and sodium channels, respectively [4].

Two case reports in the literature explain the effect of betamethasone causing hypokalaemia paralysis in pregnant women. One case developed weakness of the extremities 16 hours after the first dose, which worsened after the second dose, treated with enteral and parenteral potassium chloride. The other had worsening leg weakness after the first dose of betamethasone, managed with intravenous potassium correction [5-6]. However, in our case, the weakness was primarily due to familial hypokalaemia and would have been aggravated due to steroid injection, which was given anticipating preterm delivery. The pathophysiology behind steroid-induced hypokalaemia could be either increased renal potassium clearance or transcellular potassium shift due to secondary hyperinsulinemia.

Many precipitating factors can induce a paralytic attack, including refraining after a strenuous exercise, food with more sugar content, caffeine, exposure to cold air, fever with dehydration, or viral infection of the respiratory tract [7]. Difficulty in standing or walking in such a situation is due to decreased tone and power of the muscles, and a diminished deep tendon reflex is a classical feature: the duration, frequency, and severity of the weakness vary [8].

Treatment includes enteral or parenteral potassium supplements depending on the severity of hypokalaemia. Intravenous administration can be reserved for patients with severe disease or during the intrapartum period. Proper monitoring of the cardiac status and serum potassium level (four to six hourly) during therapy is mandatory for dose titration. Intravenous administration of potassium through media such as normal saline, ringer lactate, or mannitol can be used rather than dextrose-containing fluids as it triggers an attack [2,9]. The choice of continuing oral potassium in the antenatal or postnatal period should be individualized. An adequate intake of potassium, 4g/day during pregnancy and 4.4g/day during lactation, is required [10]. The follow-up duration can be made monthly once the serum potassium is non-fluctuating.

The mode of delivery should be individualized based on obstetric indications. Since intrapartum is a stressful period, an increase in serum catecholamine levels can provoke a paralytic episode. It can be prevented by providing labour analgesia and using operative vaginal delivery techniques in the second stage of labour to avoid increased maternal effort. However, one should be aware that there can be a clinical picture resembling malignant hyperthermia if general anaesthesia is administered [11].

## Conclusions

Though f-hypo-PP was strongly suspected due to positive family history, steroid-induced hypokalaemia could aggravate the underlying condition. Hence, steroids are better avoided in symptomatic hypokalaemia. Lifelong potassium supplementation and follow-up should be advised. A timely diagnosis and management, avoiding precipitating factors, and preventing future attacks should be the primary goal of management.
